# Supplementary material for: A deep reinforcement based echo state network for network intrusion classification
Source: PLoS One. 2026 Apr 16;21(4):e0333038. doi: 10.1371/journal.pone.0333038 (PMC13086351; doi:10.1371/journal.pone.0333038)
Supplement: Attachment — Submitted filename: Response to Reviewers - PONE-D-25-48877.pdf [file pone.0333038.s001.pdf]

Editor's Comments:

1. Please ensure that your manuscript meets PLOS ONE's style requirements, including those for file naming. The PLOS ONE style templates can be found at

https://journals.plos.org/plosone/s/file?id=wjVg/PLOSOne_formatting_sample_main_body.pdf
and
https://journals.plos.org/plosone/s/file?id=ba62/PLOSOne_formatting_sample_title_authors_affiliations.pdf

2. Please update your submission to use the PLOS LaTeX template. The template and more information on our requirements for LaTeX submissions can be found at http://journals.plos.org/plosone/s/latex.

**Author's response:** We have used the template based on the links you provided.

3. When completing the data availability statement of the submission form, you indicated that you will make your data available on acceptance. We strongly recommend all authors decide on a data sharing plan before acceptance, as the process can be lengthy and hold up publication timelines. Please note that, though access restrictions are acceptable now, your entire data will need to be made freely accessible if your manuscript is accepted for publication. This policy applies to all data except where public deposition would breach compliance with the protocol approved by your research ethics board. If you are unable to adhere to our open data policy, please kindly revise your statement to explain your reasoning and we will seek the editor's input on an exemption. Please be assured that, once you have provided your new statement, the assessment of your exemption will not hold up the peer review process.

**Author's response:** We have added the source of the dataset in **a separate new section named "Data Source"**. Previously, we have added the references of the dataset, which is why it was kept unchanged.

4. We note that Figure 1 in your submission contain copyrighted images. All PLOS content is published under the Creative Commons Attribution License (CC BY 4.0), which means that the manuscript, images, and Supporting Information files will be freely available online, and any third party is permitted to access, download, copy, distribute, and use these materials in any way, even commercially, with proper attribution. For more information, see our copyright guidelines: http://journals.plos.org/plosone/s/licenses-and-copyright. We require you to either (1) present written permission from the copyright holder to publish these figures specifically under the CC BY 4.0 license, or (2) remove the figures from your submission.

**Author's response:** We have removed the figure 1 as due to submitting revised manuscript time constraints, we are unable to reach the Flaticon. However, our methodology is written in such a way readers will not require a high level methodology figure.

Reviewer #1: Major Concerns

1. Novelty and Contribution (Critical)

ESN integration lacks theoretical justification over LSTM/GRU

Contribution claims overlap with existing DRL-based IDS work [14, 15, 20]

**Author's response:** We included theoretical justification of using ESN in Section 4.3.3. Regarding the contribution claim, the author acknowledges DRL based IDS work in [14,15,20] contains similar contributions. However, we proposed a lightweight DRL agent which can predict unseen attacks shown in section 5. Yes, benchmark models like LSTM can be more accurate than proposed ESN, but LSTM does require huge computational overhead. Our goal was to reach similar LSTM level performance by considering less computational units.

2. Zero-day attack evaluation (Table 3) is unclear—how does excluding DoS from training prove adaptive modeling when other DoS variants exist?

No computational complexity analysis despite claiming ESN is "lightweight"

Missing real-time latency measurements.

**Author response:** Our evaluation in Table 3 is a targeted simulation of real-world zero-day conditions, designed to test the model's ability to recognize novel attack variants, not merely unfamiliar categories. By training on a subset of DoS behaviors (e.g., DDoS) and testing on withheld DoS subtypes (e.g., GoldenEye), we force the model to learn the fundamental, transferable features of an attack class rather than overfitting to specific signatures. This intra-category generalization is a stricter and more meaningful test for a zero-day classifier: if a model cannot recognize a new variant of a known threat type (e.g., a novel DoS technique), it will certainly fail against entirely novel threat categories. Therefore, this split strategy directly evaluates the adaptive, feature-based reasoning essential for detecting true zero-day attacks in evolving network environments.

Thank you for the valuable feedback regarding computational complexity. You are correct that a thorough analysis strengthens the claim of being "lightweight." While real-time latency can vary significantly based on hardware, software stack, and system load making cross-study comparisons difficult, we have provided a more standardized and hardware-agnostic metric: FLOPs (Floating Point Operations) in table 10.

3. Reward mechanism (+1/-1) overly simplistic—no justification

**Author response:** The core contribution of our work is the novel application of an RL-driven model adaptation framework to the zero-day IDS problem. The simple reward function allows us to isolate and demonstrate the efficacy of this framework itself, without conflating results with the effects of a highly tuned reward signal. The strong performance achieved (as shown in our results) validates that this simple signal is sufficient for the agent to learn an effective policy for our task.

4. DRL training convergence criteria vague.

**Author's response:** Thank you for your insightful comment regarding the need for explicit convergence criteria. In response to your feedback, we have added a dedicated subsection in the methodology that details the empirical basis for our training duration and convergence assessment. Our DRL agent was trained for a fixed number of episodes (EPISODES = 31), a value determined through careful observation of training dynamics, where both reward signals and validation accuracy consistently plateaued after approximately 15–20 episodes with negligible subsequent improvement. This conservative episode limit ensures stable convergence while highlighting the sample efficiency of our ESN-enhanced architecture, which achieves robust policy optimization rapidly. We believe this clarification strengthens the reproducibility and methodological transparency of our work.

**Reviewer #2:**

1. Based on the results reported in Table 1, the proposed method does not demonstrate a clear improvement over the approaches presented in [15] and [19]. The authors are encouraged to more clearly highlight the novelty and advantages of their method compared to these existing works.

**Author response:** Regarding the contribution claim, the author acknowledges DRL based IDS work in [15,19] contains similar contributions. However, we proposed a lightweight DRL agent which can predict unseen attacks shown in section 5. Yes, benchmark models like LSTM can be more accurate than proposed ESN, but LSTM does require huge computational overhead. Our goal was to reach similar LSTM level performance by considering less computational units. We have added FLOPs score table and provided an explanation to how our proposed model is better in terms of accuracy-computational efficiency trade off.

2. The manuscript appears to be excessively long relative to the level of technical novelty it offers. A substantial portion of the paper is devoted to revisiting fundamental concepts of Deep Reinforcement Learning (DRL), which would be more appropriate for a technical report or tutorial-style paper rather than a research-oriented scientific article. The authors are advised to significantly streamline the background section and focus more on the original contributions.

**Author response:** Our writing style was intentionally tailored to align with the multidisciplinary and accessibility-focused ethos of PLOS ONE, which explicitly encourages manuscripts to be understandable to researchers across fields. Given that our work sits at the intersection of network security, machine learning, and reinforcement learning, we included a thorough yet

concise background on DRL to ensure that readers from any of these disciplines can fully engage with our contributions without needing to consult external tutorials. That said, we recognize the importance of conciseness in research articles. We made changes according to the reviewer's feedback. We believe the adjustments preserve the manuscript’s clarity for a broad audience.

3. To improve the reproducibility of the proposed approach, the authors are strongly encouraged to include a detailed description of the DRL network architecture employed in the study, such as the network structure, key hyperparameters, and training configuration.

**Author response:** Thank you for emphasizing the importance of reproducibility. We appreciate your feedback and would like to clarify that the detailed description of the DRL network architecture, including the network structure, key hyperparameters, and training configuration, was already provided in Sections 4 of the submitted manuscript.
